# Regularity of self-reported daily dosage of mood stabilizers and antipsychotics in patients with bipolar disorder

**DOI:** 10.1186/s40345-018-0118-8

**Published:** 2018-05-01

**Authors:** Maximilian Pilhatsch, Tasha Glenn, Natalie Rasgon, Martin Alda, Kemal Sagduyu, Paul Grof, Rodrigo Munoz, Wendy Marsh, Scott Monteith, Emanuel Severus, Rita Bauer, Philipp Ritter, Peter C. Whybrow, Michael Bauer

**Affiliations:** 1 Department of Psychiatry and Psychotherapy, Medical Faculty, University Hospital Carl Gustav Carus, Technische Universität Dresden, Fetscherstr. 74, 01307 Dresden, Germany; 2 ChronoRecord Association Inc., Fullerton, CA USA; 3 Department of Psychiatry and Behavioral Sciences, Stanford School of Medicine, Palo Alto, CA USA; 4 Department of Psychiatry, Dalhousie University, Halifax, NS Canada; 5 Department of Psychiatry, University of Missouri Kansas City School of Medicine, Kansas City, MO USA; 6 Mood Disorders Center of Ottawa, University of Toronto, Toronto, Canada; 7 Department of Psychiatry, University of California San Diego, San Diego, CA USA; 8 Department of Psychiatry, University of Massachusetts, Worcester, MA USA; 9 Michigan State University College of Human Medicine, Traverse City Campus, Traverse City, MI USA; 10 Department of Psychiatry and Biobehavioral Sciences, Semel Institute for Neuroscience and Human Behavior, University of California Los Angeles (UCLA), Los Angeles, CA USA

**Keywords:** Bipolar disorder, Mood stabilizers, Second generation antipsychotics, Polypharmacy, Adherence

## Abstract

**Background:**

Polypharmacy is often prescribed for bipolar disorder, yet medication non-adherence remains a serious problem. This study investigated the regularity in the daily dosage taken of mood stabilizers and second generation antipsychotics.

**Methods:**

Daily self-reported data on medications taken and mood were available from 241 patients with a diagnosis of bipolar disorder who received treatment as usual. Patients who took the same mood stabilizer or second generation antipsychotic for ≥ 100 days were included. Approximate entropy was used to determine serial regularity in daily dosage taken. Generalized estimating equations were used to estimate if demographic or clinical variables were associated with regularity.

**Results:**

There were 422 analysis periods available from the 241 patients. Patients took drugs on 84.4% of days. Considerable irregularity was found, mostly due to single-day omissions and dosage changes. Drug holidays (missing 3 or more consecutive days) were found in 35.8% of the analysis periods. Irregularity was associated with an increasing total number of psychotropic drugs taken (p = 0.009), the pill burden (p = 0.026), and the percent of days depressed (p = 0.049).

**Conclusion:**

Despite low missing percent of days, daily drug dosage may be irregular primarily due to single day omissions and dosage changes. Drug holidays are common. Physicians should expect to see partial adherence in clinical practice, especially with complex drug regimens. Daily dosage irregularity may impact the continuity of drug action, contribute to individual variation in treatment response, and needs further study.

## Background

Drug regimens for the treatment of bipolar disorder are increasing in complexity. Polypharmacy, defined as two or more psychotropic medications, is prescribed to the majority of inpatients and outpatients, including the elderly (Weinstock et al. 2014; Bjørklund et al. 2016; Bauer et al. 2013a; Peselow et al. 2016; Kleimann et al. 2016; Golden et al. 2017; Rej et al. 2017; Kessing et al. 2016). A minority of patients, ranging from 18 to 36% in recent studies, are prescribed four or more psychotropic medications (Bauer et al. 2013a; Weinstock et al. 2014; Golden et al. 2017; Goldberg et al. 2009). The use of evidence-based combination therapies may improve treatment response, and many drug combinations are included as first and second-line recommendations in international guidelines for the treatment of bipolar disorder (Parker et al. 2017; Fountoulakis et al. 2017). The challenges of polypharmacy include unproven combination therapies, limited available evidence, increased risks of serious adverse reactions and drug interactions, and patient costs (Kukreja et al. 2013; Sachs et al. 2014).

Less than half of patients with bipolar disorder are estimated to be adherent with prescribed treatments, with most having intermittent or partial adherence that fluctuates over time (García et al. 2016; Pompili et al. 2009; Scott and Pope 2002; Kessing et al. 2007). Medication non-adherence in bipolar disorder is associated with an increased risk of relapse, hospitalization, and suicide (Hassan and Lage 2009; Hong et al. 2011; Gonzalez-Pinto et al. 2006; Schuepbach et al. 2008). Medication adherence is difficult to measure, and all methods have strengths and weaknesses (Hawkshead and Krousel-Wood 2007; Pearson et al. 2007; Levin et al. 2015; Sajatovic et al. 2010). Clinical studies of adherence generally involve subjective scales completed by patients or physicians, which quantify missing days, doses or attitudes (Pompili et al. 2009; Baldessarini et al. 2008; Jónsdóttir et al. 2010). We previously measured the regularity in the daily dosage taken of mood stabilizers (lithium and anticonvulsants) using self-reported data (Bauer et al. 2013b). Dosage regularity measures daily changes and enhances understanding beyond the basic percent of missing days. Considerable irregularity in the daily dosage of mood stabilizers was found in patients missing less than 14% of days (Bauer et al. 2013b). Both the total number of psychotropic medications and the pill burden were associated with increased irregularity. The purpose of this study is to repeat the regularity analysis including second-generation antipsychotics as well as traditional mood stabilizers.

## Methods

All data were obtained from outpatients, aged 18 years or older, who agreed to record mood, sleep, and medications taken daily using ChronoRecord software (Bauer et al. 2004, 2008). All the participants were volunteers, primarily recruited by the prescribing psychiatrist, who were informed about the study prior to providing written informed consent. The study was approved by local institutional review boards. The diagnosis was made by the prescribing psychiatrist at a clinical interview, and the patient received pharmacological treatment as usual throughout the study. Data were obtained from 666 patients, of which 480 had a diagnosis of any bipolar disorder based on DSM IV or DSM 5 criteria, and returned ≥ 30 days of data.

### Patient data entry

Patents received about a half hour of training on the use of ChronoRecord software, in person or by telephone, before entering data. During the training session, a medication list was created for each patient. The medication list includes all drugs prescribed for bipolar disorder and any other prescribed or over-the-counter (OTC) drugs that the patient felt impacted their mood. The prescribed psychotropic drugs were selected from a list in the software, displayed by both brand and generic names. The patient could add a drug not included in the software list, and could modify the list of drugs taken at any time. For each selected drug, the pill strength was chosen from a list of available strengths. Every day, for each drug, the patient entered the total number of pills taken. Patients could enter partial pills (1/4, 1/2, or 3/4) for tablets but not capsules. If a drug was not taken, the patient entered 0 pills for that drug for that day. A missing day of data was also treated as if no pills were taken. Data not entered on 1 day could be entered at a later date. The software includes error checking to prevent entry for a future date, and to verify entry of a large number of pills for a drug.

In addition to medications, the patients entered mood, sleep, and significant life events daily, and weight weekly into the ChronoRecord software. ChronoRecord uses a 100-unit visual analog scale between the extremes of mania and depression to rate mood. Based upon the prior validation studies (Bauer et al. 2004, 2008), a mood entry less than 40 was considered depression, 40–60 euthymia, and greater than 60 hypomania/mania. The depression ratings varied from mild (entry of 20–39) to moderate–severe (entry of 0–19), and the mania ratings varied from hypomania (entry of 61–80) to moderate-severe (entry of 81–100).

### Drugs analyzed

The drugs analyzed were traditional mood stabilizers as in the prior analysis: lithium, valproate, lamotrigine, carbamazepine, oxcarbazepine (Bauer et al. 2013b), and second generation antipsychotics: aripiprazole, olanzapine, risperidone, quetiapine, ziprasidone, paliperidone, asenapine, lurasidone, and clozapine. The analysis of the total psychotropic drugs taken and the daily pill burden also included antidepressants, benzodiazepines, typical antipsychotics, insomnia medications, other anticonvulsants (topirimate, gabapentin, pregabalin, tiagabine, levetiracetam, zonisamide), thyroid hormones and estrogens.

### Regularity analysis using ApEn

Regularity in the daily medication dosage was calculated using Approximate Entropy (ApEn) as in the prior studies (Bauer et al. 2013b, c). ApEn is a family of statistics that measure serial regularity in a time series, are model independent, and can be used with datasets that are small and noisy (Pincus 1991; Pincus et al. 1991). Regularity can be thought of as the tendency that values within a time series remain the same on incremental comparisons (Pincus 1991; Pincus et al. 1991). ApEn computes a single positive value, with 0 indicating a completely regular sequence, and with increasingly larger numbers signifying greater serial irregularity. The estimated value of the ApEn (m, r, N) depends on: m the pattern length used for prediction of the subsequent value, r the level of noise filtering, and N the number of data values in the run to be compared. The ApEn noise filtering compares the difference between the data in each sequence of m days of data (Pincus et al. 1991). Conceptually, when the absolute value of the difference is greater than the noise filter level, the ApEn statistic is incremented. Otherwise, the difference is treated as noise and ignored. The level of noise filtering was calculated as a percent of the individual subjects’ standard deviation (Pincus et al. 1993). For this analysis, the parameters included were m = 1 day, r = 0.2 × SD in daily drug dosage, and N = 100 days.

The calculated value of ApEn is dependent upon the order of the data in the time series. Changing the order of the data will likely change the calculated value of ApEn. In contrast, the calculated value of the familiar mean and standard deviation will be identical for a set of data in a time series regardless of the order of the data. ApEn is a valuable measure of the daily dosage fluctuations found with partial adherence (Bauer et al. 2013b, c). For example, the ApEn would be 0 if the patient made no changes to the daily dosage or discontinued a medication, and would be largely unaffected by a prescription change if the patient keeps taking the new dosage (Bauer et al. 2013b).

### Per patient regularity analysis

For every drug included in the ApEn analysis, the time span for taking the drug was determined for each patient. If the time span was ≥ 100 days, the ApEn was calculated for the first 100 days of data. Patients who took more than one drug for ≥ 100 days could have more than one ApEn analysis. Of the 480 patients with bipolar disorder, 241 patients took at least one drug for sufficient length of time for analysis. For the 241 patients, 422 ApEn sequences were calculated.

### Statistics

Descriptive statistics for the demographic and clinical characteristics of the 241 patients were calculated. For each patient, for each 100-day ApEn period, the percent of days with depressed, euthymic and manic/hypomanic mood were determined. Drug holidays, defined as missing 3 or more consecutive days (Urquhart 1998), were determined for each patient during the 100-day period. The daily pill burden was defined as total number of pills for all psychotropic medications. For each patient, for each 100-day ApEn period, the mode of the daily number of psychotropic medications, daily pill burden, and daily dosage were calculated. The mode is the most frequent value in a series of numbers, and was chosen as a proxy for the prescribed daily number of medications, pill burden and daily dosage. The mean values for the entire sample were calculated using the modal values for each patient.

Since one patient could have more than one ApEn analysis, generalized estimating equations (GEE) were used to adjust model coefficients and standard errors for within-patient correlation. To estimate if demographic or clinical variables were associated with ApEn, GEE models were used with ApEn as the dependent variable and an independent working correlation structure (Pan and Connett 2002). GEE models were also used to estimate if demographic or clinical variables were associated with the percent of missing doses or with drug holidays. SPSS Version 24 was used for all analyses.

## Results

422 ApEn sequences were calculated from the 241 patients. Of the 241 patients, 158 (66%) were recruited from a university mood clinic and 83 (34%) from a private practice. The demographic characteristics of the 241 patients are shown in Table 1. During the 100-day periods, the patients were euthymic 71.6% of days, depressed 20.9% of days, and hypomanic/manic 7.5% of days. The 241 patients returned a mean of 389 (SD 567) days of data.

**Table 1:** Patient demographics (N = 241)

Demographic	N	%
Gender (N = 242)		
Male	70	29
Female	171	71
Diagnosis (N = 240)		
BP I	145	60
BP II	86	36
BP NOS	9	4
Marital status (N = 223)		
Married	111	50
Divorced	29	13
Single	83	37
Employment status (N = 207)		
Working full-time	94	45
Disabled	52	25
Other	61	30
Education (N = 225)		
High school	29	13
Some college	72	32
College graduate	124	55
	*Mean*	*SD*
Age (N = 241)	41.2	10.9
Age of onset (N = 225)	22.6	10.5
Hospitalizations (N = 218)	2.8	4.7
Years of illness (N = 225)	18.9	12.1

### Medications

The medications taken by the 241 patients are summarized in Table 2. The patients took a mean of 3.9 psychotropic medications, with a mean pill burden of 7.2 for these drugs. Many of the 241 patients were taking more than one mood stabilizer or antipsychotic, or changed medication, such that 121 (50%) of the patients had 1 ApEn analysis, 72 (30%) had 2 ApEn analysis, 40 (17%) had 3 ApEn analyses, and 8 (3%) had > 3 ApEn analyses.

**Table 2:** Psychotropic medications taken during the 100-day analyses periods (N = 241)

Medication	N	%		
Taking antidepressants	122	51		
Taking benzodiazepines	55	23		
Taking insomnia medications	22	9		

All medications^a^	Mean	SD		
Total number of medications	3.9	2.0		
Total pill burden	7.2	4.8		

Antipsychotic/mood stabilizer^b^	N in analysis	Mean dosage (mg)	Dosage SD	Pct days missing
Aripiprazole	28	15.5	11.3	15.4
Risperidone	17	1.6	1.4	19.5
Quetiapine	48	268.4	202.0	22.3
Ziprasidone	12	141.6	59.4	12.9
Olanzapine	17	8.4	5.4	13.5
Lithium	99	914.6	320.6	15.1
Valproate	45	1107.2	599.0	12.8
Carbamazepine	15	798.1	386.1	5.1
Oxcarbazepine	14	938.1	605.6	26.1
Lamotrigine	119	231.9	129.4	13.5

^a^Only psychotropic drugs

^b^Only including drugs with ≥ 10 analysis periods

### Missing days and drug holidays

Overall, the patients took medication on a mean of 84.4% of days. Missing drug data occurred frequently within the 100-day analysis periods. There was at least one single day omission in 64.7% of the 422 analyses periods. The percent of days of missing drug data was associated with the percent of days depressed (p = 0.046), and inversely associated with the percent of days euthymic (p = 0.047).

One or more drug holidays were found in 151 (35.8%) of the 422 analysis periods. Of the 151 analysis periods containing a drug holiday, more than one drug holiday was present in 57 (37.8%). Taking a drug holiday was associated with working full time (p = 0.005), and the total number of psychotropic drugs (p = 0.043).

### Regularity analysis

For the 422 100-day analyses periods, the ApEn values ranged between 0 and 0.94, with a mean of 0.21 (SD 0.18). For the 422 analyses, the ApEn was between 0 and 0.2 for 240 (56.9%), between 0.2 and 0.4 for 128 (30.3%), and > 0.4 for 54 (12.8%). The ApEn was 0 (no change to daily dosage) in 56 (13.3%) of the analyses.

**Fig. 1:**
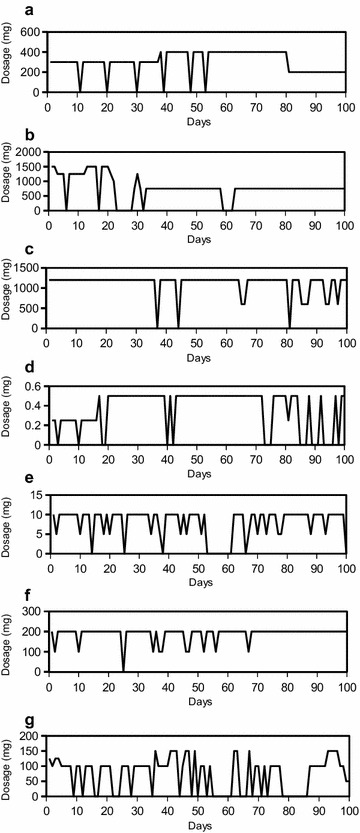
Example ApEn, adherence and drug holiday values for 100 days of data. In **a**–**f**, patients have few missing days but irregular daily dosage (high ApEn). In **g**, the patient has both a large number of missing days and irregular daily dosage. **a** Lamotrigine. ApEn 0.3432 with 94% adherence and 0 drug holidays. **b** Valproate. ApEn 0.4836 with 87% adherence and 2 drug holidays. **c** Lithium. ApEn 0.3511 with 97% adherence and 0 drug holidays. **d** Risperidone. ApEn 0.6007 with 80% adherence and 4 drug holidays. **e** Asenapine. ApEn 0.7117 with 86% adherence and 1 drug holiday. **f** Lamotrigine. ApEn 0.3756 with 99% adherence and 0 drug holidays. **g** Lamotrigine. ApEn 0.9428 with 65% adherence and 4 drug holidays

ApEn is directly related to the percent of days of missing doses (p < 0.001). However, even patients with a low percent of days missing doses may have irregular daily dosages. Figure 1 includes examples graphs of patients with irregular daily dosage, despite low missing days of data, and one example of how a patient may have both a larger number of missing days, and variable dosage.

The factors other than missing days of data that were associated with increasing irregularity are shown in Table 3. The total number of psychotropic drugs (p = 0.009), the pill burden (p = 0.026), and the percent of days depressed (p = 0.049) were all associated with irregularity. The percent of days euthymic was inversely related to irregularity (p = 0.028). No other clinical or demographic variables were associated with irregularity.

**Table 3:** Estimated coefficients of parameters associated with daily dosage irregularity for mood stabilizers and second generation antipsychotics^a^

Parameter	Coefficient estimate	Standard error	95% Wald confidence interval		Coefficient significance	
			Lower	Upper	Wald chi square	p
Total pill burden^b^	0.005	0.002	0.001	0.010	4.944	0.026
Total number of medications^b^	0.015	0.006	0.004	0.027	6.771	0.009
Percent days depressed	0.001	0.001	4.137E−6	0.003	3.867	0.049
Percent days euthymic	− 0.001	0.001	− 0.002	0.000	4.845	0.028
Percent days manic	0.001	0.001	− 0.001	0.003	1.870	0.171

^a^(422 100-day analyses periods). GEE model estimated ApEn (1, 0.2  * SD, 100) using the listed parameter with an independent working correlation structure for each model

^b^Psychotropic medications only

## Discussion

Patients with bipolar disorder in this study were motivated to actively participate in their care, and took medication on about 84% of days. Yet, even among this group, despite the low percent of missing days, there was considerable irregularity in the daily dosage taken. Days of missing doses, primarily single day omissions, and changes to the daily dosage were the primary cause of the irregularity. Additionally, there was at least one drug holiday in 35.8% of the analysis periods. These results are consistent with our prior studies, and there are many implications of these findings (Bauer et al. 2013b, c).

It is challenging for the physician to reliably assess patient adherence and the link between non-adherence and inadequate response. Irregularity in the daily dosage may be a contributing factor. Most psychiatrists prefer to assess adherence of patients with bipolar disorder by asking the patient (Vieta et al. 2012), but physician perceptions are often incorrect and optimistic (Velligan et al. 2009; Baldessarini et al. 2008). For example, physicians overestimated adherence with second-generation antipsychotics, primarily by patients with bipolar disorder, as compared to claims data (Stephenson et al. 2012). For 97 patients who went to an emergency room for an exacerbation of psychosis, including 26 with bipolar disorder, staff assessments of adherence and non-adherence were correct 41.5 and 75% of the time respectively, when compared to plasma antipsychotic levels (Lopez et al. 2017).

In this study, single day omissions occurred frequently. Some drugs and formulations are more forgiving about dosage omissions than others, varying with the pharmacokinetic and pharmacodynamic properties (Osterberg et al. 2010; Urquhart 1998). With a forgiving drug, the duration of action is much longer than the dosage interval, so an occasional missed dose is unlikely to interrupt therapeutic activity (Osterberg et al. 2010). Recently, the number of drug formulations that require less frequent dosing has increased, including formulations for psychotropic drugs. While a less frequent dosing regimen generally increases adherence (Saini et al. 2009; Claxton et al. 2001), it may not improve outcomes (Comté et al. 2007; Richter et al. 2003; Vrijens et al. 2014; Vrijens and Heidbuchel 2015; Harden 2017; Bialer 2007). For example, the consequences of missing one dose of a once-daily drug may be more deleterious to the continuity of therapeutic action than missing one dose of a twice-daily drug (Osterberg et al. 2010; Hughes 2006; Urquhart and Vrijens 2006). However, extended release formulations that reduce fluctuations in plasma concentration may improve the forgiveness of once-daily drugs (Pellock and Brittain 2016; Chen et al. 2013; Brittain and Wheless 2015).

The relatively large number of drug holidays reported in this study, with a dosing interruption of 3 or more days, are of considerable concern. The rapid discontinuation of a psychotropic drug may trigger immediate withdrawal symptoms or delayed rebound phenomena, related to complex factors including pharmacokinetic and pharmacodynamic properties of a drug formulation, and individual metabolism (Baldessarini et al. 1999; Cerovecki et al. 2013; Franks et al. 2008; Fava et al. 2015; Correll 2010; Osterberg et al. 2010). After a drug holiday, patients often resume taking the full-strength dosage, including of drugs that are slowly titrated upward. Re-starting a drug after a long lapse may trigger first-dose effects (Urquhart 1998). Given the frequent use of polypharmacy, drug holidays may impact the potential for drug interactions (Spina et al. 2016). There is a need for increased understanding of the clinical impacts of repeated starting and stopping of mood stabilizers and antipsychotic drugs, in various product formulations (Osterberg et al. 2010; Hughes 2008; Samtani et al. 2012). In this study, taking a drug holiday was associated with working full time, suggesting that some patients may doubt they need ongoing treatment (Clatworthy et al. 2009), or lack insight into the value of medications (Copeland et al. 2008). In prior research, employment was not associated with psychotropic medication adherence (Bulloch and Patten 2010; Razzano et al. 2005, Sajatovic et al. 2006).

In addition to dosage omissions, changes to the daily dosage contributed to the irregularity. The patients in this study took polypharmacy with a mean of 3.9 psychotropic medications for bipolar disorder, and a mean pill burden of 7.2. Both the number of psychotropic medications and the pill burden were associated with irregularity in daily dosage. Research in a wide range of chronic medical illnesses has found that medication regimen complexity decreases adherence (Ingersoll and Cohen 2008). Some patients with bipolar disorder may have trouble integrating a complex drug regimen into their daily routine (Sajatovic et al. 2009; Wagner and Ryan 2004), especially those with a disorganized lifestyle (Frank et al. 2006). Even patients intent on adhering often forget about doses, especially on days with unexpected schedule interruptions (Dunbar-Jacob and Mortimer-Stephens 2001; Bulloch and Patten 2010), which usually results in underdosing but sometimes overdosing.

Other factors contribute to irregular daily dosages. Patients may have an “as needed” approach to dosing, taking doses to treat symptoms or lessen side effects (Dunbar-Jacob and Mortimer-Stephens 2001; Marder 2003; Pound et al. 2005). Recent societal emphasis on self-management may be encouraging self-experimentation (Swan 2013). Some patients want to take as little medication as possible (Pound et al. 2005). Consistent with our prior studies, depressive symptoms were associated with increased irregularity (Bauer et al. 2013b, c). In other research, depressive symptoms were associated with non-adherence in bipolar disorder (Belzeaux et al. 2013; Johnson et al. 2007), as well chronic medical conditions (Grenard et al. 2011).

Several issues may impact the generalizability of this study. No data were available on the rate and characteristics of patients who were asked but declined to participate in the study. A large percentage of patients were recruited from university clinics, which may not reflect other settings. In the current study, more females than males were included, and patients varied in the phase of illness and disease severity. However, the demographic characteristics of the patients who use ChronoRecord are similar to those reported for other studies of bipolar disorder (Bauer et al. 2012). Since taking a mood stabilizer or antipsychotic for 100 days was required for analysis, the least adherent patients were excluded. However, even higher irregularity in daily dosage would be expected from patients who are less adherent.

There are other limitations to this study. All data were self-reported. However, review articles about the measurement of medication adherence have found moderate-to-high concordance between self-reported patient questionnaires and diaries, and electronic monitoring (Garber et al. 2004; Shi et al. 2010; Monnette et al. 2018). In a study of patients with bipolar disorder, good agreement was found between patient questionnaires and serum levels of psychotropic medications (Jónsdóttir et al. 2010). This study underestimated regimen complexity since medications taken for general medical reasons and OTC drugs were not included. Other aspects of drug regimen complexity such as administration instructions, and dosage timing were not available. Some of the dosage changes may have been prescribed by the physician. Only oral medications were included in this study. Different formulations of the same medication, such as pill size and ease of swallowing, are known to impact adherence but were not considered (Bhosle et al. 2009; Fields et al. 2015). The specific drug regimens were not investigated but are highly variable in clinical practice, including different combinations of medication classes and drugs (Bauer et al. 2013a).

It is important to put the findings of this study into context. In this sample of patients with bipolar disorder who were motivated to participate in their care, there was considerable irregularity in daily dosage. However, partial adherence is also routinely found in patients with chronic medical conditions who do not have mental illness (Osterberg and Blaschke 2005; Brown et al. 2016). Even in a monitored environment, less than 70% of over 16,000 patients with various medical conditions enrolled in 95 clinical studies were fully adherent (Vrijens and Urquhart 2014; Blaschke et al. 2012). The clinician treating patients with bipolar disorder should expect a level of nonadherence including dosage omissions, changes, and drug holidays, even among patients determined to recover. If the patient’s condition is such that exceptional adherence is required, such as approaching no missing dosages, intensive educational measures, and customization of the individual’s regimen are required. These findings also confirm the need for careful evaluation of newly appearing or worsening symptoms.

## Conclusion

In conclusion, considerable irregularity in daily dosage of mood stabilizers and antipsychotic medications was found despite a low percent of missing days. The total number of psychotropic drugs, pill burden and depression were associated with increased irregularity. The irregularity in daily dosage was primarily due to single day omissions and dosage changes. Drug holidays were also present. These findings may contribute to understanding the individual variation in treatment response seen in clinical practice. Physicians should expect to see partial adherence with treatments for bipolar disorder. More understanding of the impacts of daily irregularity on specific drugs and formulations is needed.
